# Correcting for Microbial Blooms in Fecal Samples during Room-Temperature Shipping

**DOI:** 10.1128/mSystems.00199-16

**Published:** 2017-03-07

**Authors:** Amnon Amir, Daniel McDonald, Jose A. Navas-Molina, Justine Debelius, James T. Morton, Embriette Hyde, Adam Robbins-Pianka, Rob Knight

**Affiliations:** a Department of Pediatrics, University of California San Diego, La Jolla, California, USA; b Department of Computer Science and Engineering, University of California San Diego, La Jolla, California, USA; c Department of Computer Science, University of Colorado Boulder, Boulder, Colorado, USA; d Center for Microbiome Innovation, University of California San Diego, San Diego, California, USA; University of Copenhagen

**Keywords:** 16S rRNA, DNA sequencing, bioinformatics

## Abstract

The use of sterile swabs is a convenient and common way to collect microbiome samples, and many studies have shown that the effects of room-temperature storage are smaller than physiologically relevant differences between subjects. However, several bacterial taxa, notably members of the class *Gammaproteobacteria*, grow at room temperature, sometimes confusing microbiome results, particularly when stability is assumed. Although comparative benchmarking has shown that several preservation methods, including the use of 95% ethanol, fecal occult blood test (FOBT) and FTA cards, and Omnigene-GUT kits, reduce changes in taxon abundance during room-temperature storage, these techniques all have drawbacks and cannot be applied retrospectively to samples that have already been collected. Here we performed a meta-analysis using several different microbiome sample storage condition studies, showing consistent trends in which specific bacteria grew (i.e., “bloomed”) at room temperature, and introduce a procedure for removing the sequences that most distort analyses. In contrast to similarity-based clustering using operational taxonomic units (OTUs), we use a new technique called “Deblur” to identify the exact sequences corresponding to blooming taxa, greatly reducing false positives and also dramatically decreasing runtime. We show that applying this technique to samples collected for the American Gut Project (AGP), for which participants simply mail samples back without the use of ice packs or other preservatives, yields results consistent with published microbiome studies performed with frozen or otherwise preserved samples.

**IMPORTANCE** In many microbiome studies, the necessity to store samples at room temperature (i.e., remote fieldwork) and the ability to ship samples without hazardous materials that require special handling training, such as ethanol (i.e., citizen science efforts), is paramount. However, although room-temperature storage for a few days has been shown not to obscure physiologically relevant microbiome differences between comparison groups, there are still changes in specific bacterial taxa, notably, in members of the class *Gammaproteobacteria*, that can make microbiome profiles difficult to interpret. Here we identify the most problematic taxa and show that removing sequences from just a few fast-growing taxa is sufficient to correct microbiome profiles.

## Abstract (precis)

In many microbiome studies, the necessity to store samples at room temperature (i.e., remote fieldwork) and the ability to ship samples without hazardous materials that require special handling training, such as ethanol (i.e., citizen science efforts), is paramount. However, although room-temperature storage for a few days has been shown not to obscure physiologically relevant microbiome differences between comparison groups, there are still changes in specific bacterial taxa, notably, in members of the class *Gammaproteobacteria*, that can make microbiome profiles difficult to interpret. Here we identify the most problematic taxa and show that removing sequences from just a few fast-growing taxa is sufficient to correct microbiome profiles.

## OBSERVATION

The use of sterile swabs is a convenient way to collect samples for microbiome studies, but in some cases, it is not feasible to immediately freeze or utilize a preservative. For example, the American Gut Project (AGP; Qiita study identifier [ID] 10317) allows members of the general public to send samples for 16S rRNA gene amplicon sequencing through domestic post without a preservative. This is because proven preservation methods can be cumbersome, dangerous, expensive, or sample type specific, complicating participation in microbiome citizen science. Although some studies have demonstrated that the effects of room-temperature storage are secondary to physiologically relevant differences between comparison groups (1–3), certain bacterial taxa, particularly those in the class *Gammaproteobacteria*, grow well at room temperature. This is problematic, as some *Gammaproteobacteria* species have been associated with disease, such as inflammatory bowel disease (IBD) (4). Therefore, to identify meaningful patterns in microbiome studies that do not utilize sample preservation, it is crucial to remove at high specificity the taxa that thrive at room temperature (i.e., “blooming” bacteria).

Here we performed a meta-analysis that combined fecal samples from storage experiments (low sample numbers but easily interpretable results) with bulk sample statistics from projects comparing room-temperature shipping to immediate freezing, identifying exact sequences corresponding to blooms by applying Deblur (5) to the data sets. We assessed whether any sequences are enriched more than expected in room temperature samples, producing a list of candidate sub-operational taxonomic units (sOTUs) or exact sequences that appear to increase in frequency at room temperature. We then filtered these exact sequences from the AGP data set, restoring a biological association that was obscured by the blooms. We further validate the procedure by confirming that filtered data sets more closely resemble those from immediately frozen samples and by showing that the overall microbiome profiles better match the results of other published human microbiome studies.

**FIG 1:**
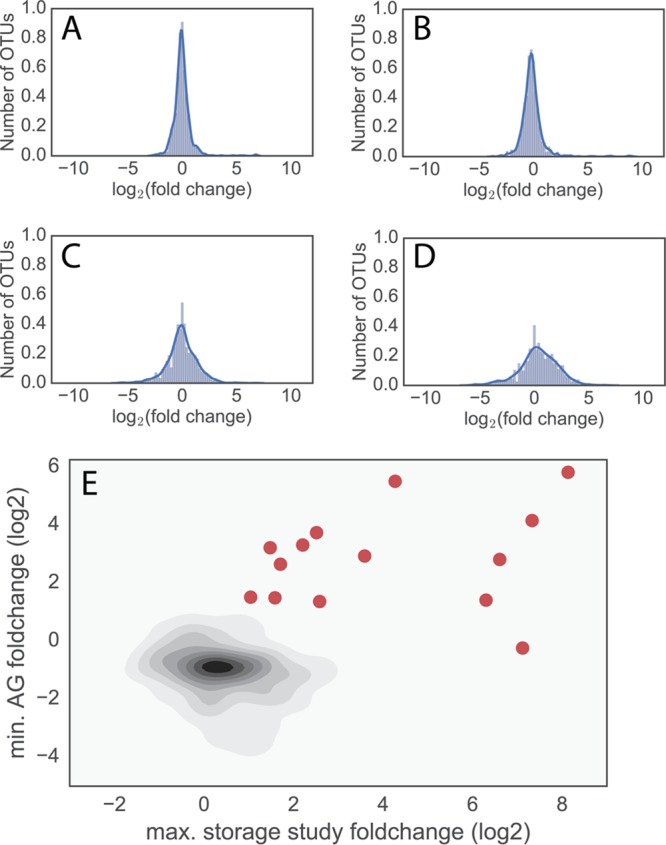
A small number of bacteria showed high levels of growth in the storage studies. (A to D) The log_2_ fold change of an OTU relative to time zero within the Mayo Clinic fecal stability study and the preservation study by Song et al. (1). A minimum read threshold of 10 reads per sample was used, and OTUs present below this threshold in both samples are not shown. (A and B) Mayo Clinic fecal stability study (2) at day 1 (A) and day 4 (B). (C and D) Results of fecal preservation study by Song et al. (1) at day 7 (C) and day 14 (D). The numbers (and percentages of total bacteria) showing at least 4-fold change in the room-temperature storage compared to day 0 were 11 (1.6%), 21 (3.1%), 49 (7.1%), and 144 (19%) for days 1, 2, 7, and 14, respectively. (E) A topology plot of the maximal (max.) fold change in the two storage studies whose results are shown in panels A to D (*x* axis) and minimal (min.) fold change in the AGP fecal samples compared to data reported in references 6 and 7 and PGP (unpublished), studies in which samples were immediately frozen (*y* axis). Red circles denote 14 bacterial samples selected as potentially blooming (>2-fold change in both axes or >50-fold change in the storage studies). Six bacterial samples showing >2-fold change in AGP compared to results of all studies using fresh-frozen samples that were not present in the storage studies are also added to the potential blooming list (Table S1).

To identify the candidate blooming bacteria, we first examined the effect of room-temperature storage on fecal microbiome samples. Using two recent storage studies (1, 2), we showed that taxonomic abundance changes over time in nonfrozen fecal samples compared to frozen samples are mainly due to a small number of taxa (Fig. 1A to D). The taxa that contributed disproportionately are primarily members of the class *Gammaproteobacteria*, which is unsurprising given that many members of this class are easily cultivable, fast-growing and are commonly isolated from human stool. Unfortunately, these storage studies examined samples from a small number of individuals, and therefore it is possible that additional bacterial taxa bloom in samples shipped via domestic post that by chance were not present in these controlled studies. To address this limitation, we compared all AGP fecal samples (~7,000 samples) to data from 3 studies comprised of fecal samples immediately frozen (fresh-frozen) after collection (6, 7; Personal Genome Project [PGP; unpublished data, Qiita study ID 1189]). Importantly, because each study represented a different population, it is likely that sOTUs were present at different frequencies across these studies. Nevertheless, blooming bacteria are expected to be at a higher frequency in AGP samples than in all of the fresh-frozen samples.

**FIG 2:**
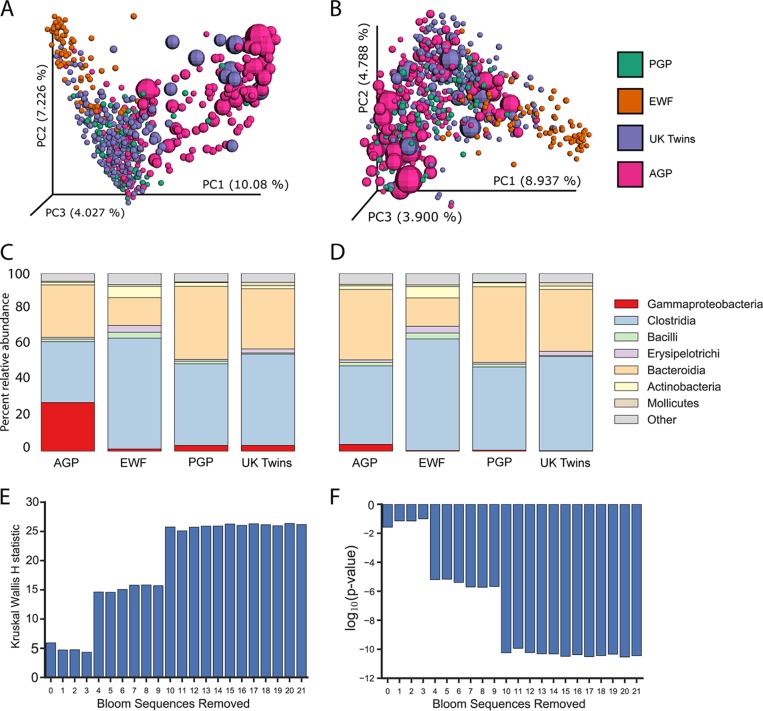
Effect of bloom filtering on American Gut data. (A and B) PCoA of Bray-Curtis distances from a random subset of 200 American Gut Project samples (AGP [unpublished]; colored pink) compared to 3 studies containing fresh-frozen fecal samples: Personal Genome Project (PGP [unpublished]; colored green); whole-grain feces (EWF [6]; colored orange); and UK Twins ([7]; colored purple), respectively. The PCoA data shown represent results obtained before (A) and after (B) applying the filter for blooms to all samples. The size of a sphere is scaled by the amount of candidate bloom bacteria in a sample prior to filtering. (C and D) Mean taxonomy distribution for the same studies before (C) and after (D) filtering for blooms. (E and F) The well-known effect of age on alpha diversity and how the effect is observed only after the removal of bloom reads. The Kruskal-Wallis test statistic (E) and corresponding –log(*P* value) (F) are shown for different numbers of bacteria used for the filtering before applying the test. A value of 0 on the *x* axis indicates no filtering. The *x* axis is ordered by decreasing severity score of the bloom where bloom 1 represents greater severity than bloom 2, and each point on the *x* axis includes the prior blooms (e.g., position 5 includes bloom sOTUs 1 through 5).

Using reasonable thresholds for relative abundance changes in the storage studies and in the AGP compared to fresh-frozen studies, we identified 20 bacterial sOTUs as candidates for blooming during shipping (Fig. 1E; see Table S1 in the supplemental material) using the following criteria: a fold increase of 2 or more in the room-temperature storage studies (1, 2) and AGP relative to fresh-frozen fecal samples from studies (6, 7; PGP) and a fold increase of 50 or more within the storage studies only or not observed in the storage studies but with at least a 2-fold change in AGP compared to the fresh-frozen studies. The results appear insensitive to these specific thresholds, as we found that removal of a subset of 10 of the identified candidate blooms from the AGP cohort was sufficient to restore a well-characterized age correlation with alpha diversity (Fig. 2E and F) and was sufficient for a significant decrease in the distances to fresh-frozen samples (see Fig. S1 in the supplemental material).

10.1128/mSystems.00199-16.2 FIG S1  Effect of number of candidate blooming bacteria used for filtering. Data represent mean Bray-Curtis distances between different experiments following removal of 0 to 20 candidate blooming bacteria (*x* axis). A total of 1,000 random pairs of samples were chosen for each level of filtering. Distances shown correspond to comparisons of American Gut to UK Twins (red), American Gut to PGP (blue), American Gut to whole-grain feces (yellow), UK Twins to PGP (dashed green), UK Twins to whole-grain feces (dashed black), and whole-grain feces to PGP (dashed light blue). Download FIG S1, PDF file, 0.02 MB. Copyright © 2017 Amir et al. 2017 Amir et al. This content is distributed under the terms of the Creative Commons Attribution 4.0 International license.

10.1128/mSystems.00199-16.3 TABLE S1  A spreadsheet containing the list of candidate blooming bacteria and their fold change, sorted by significance. Download TABLE S1, XLSX file, 0.05 MB. Copyright © 2017 Amir et al. 2017 Amir et al. This content is distributed under the terms of the Creative Commons Attribution 4.0 International license.

To mitigate the effect of these blooming bacteria on subsequent microbiome analyses, we removed exact sequence matches to identified blooms from 35,146 unique sOTUs identified by applying Deblur (5) to 10,189 samples spanning 338,496,967 sequences from the AGP data set. Each of the 20 blooms had an exact match to one of the unique Deblur sOTUs, and a total of 32,696,826 reads were removed (per-sample dropped sequences spanning 0.4%, 13.1%, and 45.3% for the 25th, 50th, and 75th percentiles, respectively). Importantly, some of the removed sequences were likely “real”; for example, *Escherichia coli* and *Citrobacter* sequences present in the candidate blooming list were present at nonnegligible frequencies in fresh-frozen samples. However, these sequences were included for removal as their tendency to grow during shipment can greatly impact the relative abundances of other organisms due to the compositional nature of the data.

Without filtering candidate blooms, there were notable differences (as observed using Bray-Curtis principal-coordinate analysis [PCoA]) between AGP fecal samples and the fresh-frozen fecal samples; filtering the bloom sequences from all samples removed these differences (Fig. 2A versus B). In the PCoA space corresponding to the data determined without filtering, the primary separation is explained by the presence of a large percentage of bloom sequences (Fig. 2A); the sizes of the spheres are scaled by the percentage of bloom sequences in the respective sample. Following the removal of the blooms, this dominant effect was abolished and samples with high levels of blooms clustered with samples from the other studies (Fig. 2B). Similar results were observed in assessing class-level taxonomy abundances (Fig. 2 versus D): prior to filtering, a high relative abundance of *Gammaproteobacteria* (27%) was present in the AGP samples compared to the fresh-frozen samples (1.5% to 3.5%), while the AGP profile seen after filtering more closely resembled that of the fresh-frozen samples. Importantly, applying the filter minimally changed the taxonomic profiles of fresh-frozen samples (Fig. 2D). The filtering procedure is available in a Jupyter Notebook (8) at https://github.com/knightlab-analyses/bloom-analyses.

There is a balance between type 1 and type 2 errors that must be considered in applying this filter. The cost of removing a sequence is that it becomes “invisible” in the analysis, and it is possible that real sequences are lost. Conversely, retaining a bloom sequence increases noise caused by shipment conditions, which can artificially alter biological conclusions. Therefore, a balance between loss of data and inaccurate, noisy data must be obtained. To select an appropriate number of blooming bacterial sequences to subtract from the AGP data set to maximize the amount of data retained while reducing inaccuracies caused by blooms, we tested the effect of nested filtering levels on the ability to detect the well-known effect of age on alpha diversity (9, 10). As can be seen in Fig. 2E and F, this effect was undetected by a Kruskal-Wallis test when none of the candidate blooms were removed. However, filtering the top four candidate blooms restored the ability to detect a significant difference in diversity by age. Critically, the identification of the bloom sOTUs was done independently of this positive control. For analysis of the AGP cohort, we recommend removal of the sequences of the top 10 candidate blooming bacterial taxa, as this maximizes the expected age effect (Fig. 2E). Different studies may want to remove a different subset of bloom sequences, as retaining some of these sequences might be critical, depending on the study characteristics. With meta-analysis, if this filter is applied, it must be applied identically to all samples represented to avoid introduction of a systematic bias.

Given that most bacteria change in relative abundance relatively little, filtering for blooms removes an important confounding variable and facilitates meta-analysis of projects that have used different storage procedures. We recommend this procedure to facilitate analysis of data produced from fecal studies without the means to immediately freeze or preserve samples such as citizen science efforts or remote fieldwork where it may be impossible to preserve samples immediately. Additionally, these data suggest that further control studies should be performed to allow the evaluation of candidate blooms and their impacts in nonfecal environments.

10.1128/mSystems.00199-16.1 TEXT S1  Details on materials and methods. Download TEXT S1, DOCX file, 0.1 MB. Copyright © 2017 Amir et al. 2017 Amir et al. This content is distributed under the terms of the Creative Commons Attribution 4.0 International license.
