# Supplementary figures and images for: Correcting for Microbial Blooms in Fecal Samples during Room-Temperature Shipping
Source: mSystems. 2017 Mar 7;2(2):e00199-16. doi: 10.1128/mSystems.00199-16 (PMC5340865; doi:10.1128/mSystems.00199-16)

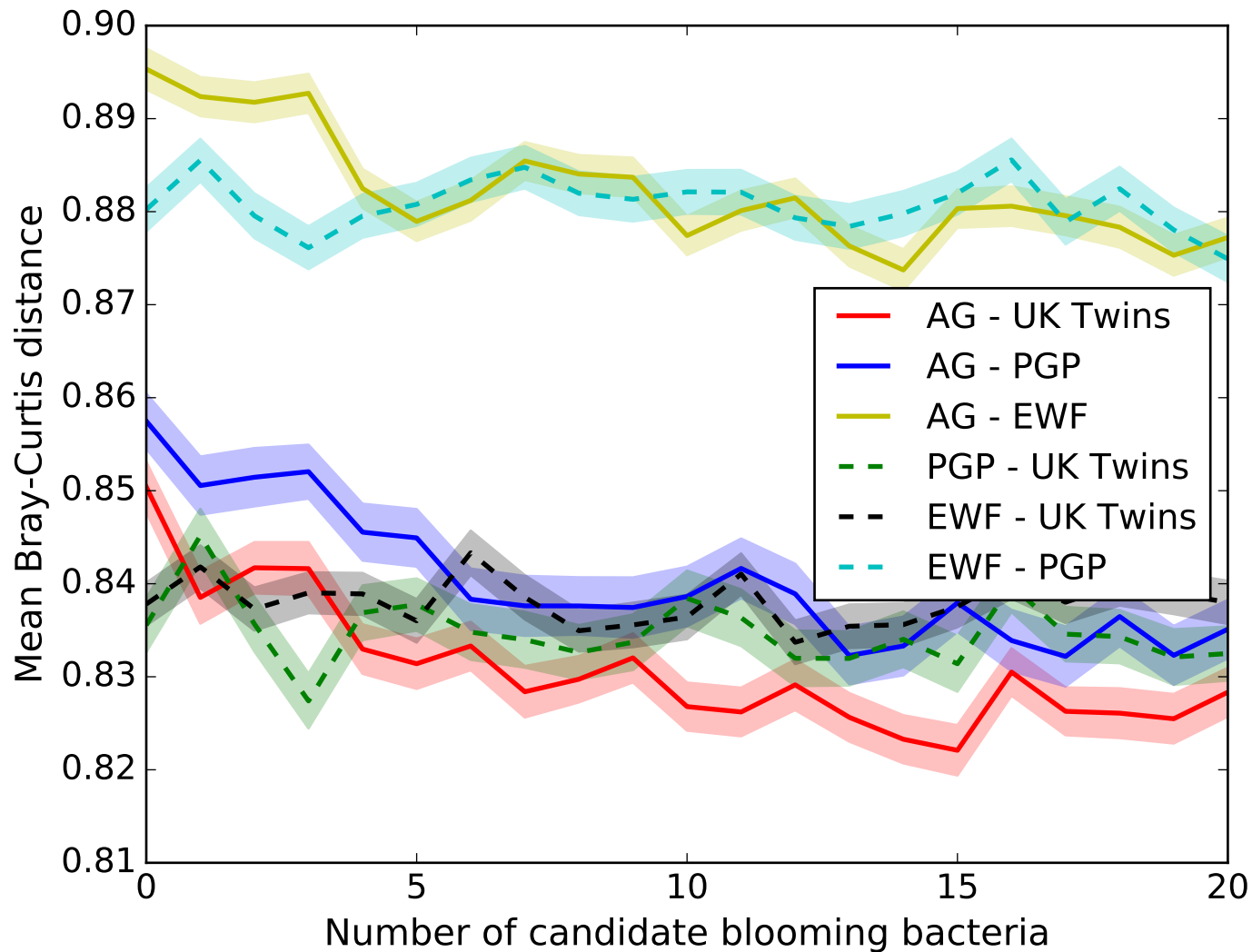

Supplement: FIG S1 [file sys002172095sf2.pdf]
