# Supplementary material for: Correcting for Microbial Blooms in Fecal Samples during Room-Temperature Shipping
Source: mSystems. 2017 Mar 7;2(2):e00199-16. doi: 10.1128/mSystems.00199-16 (PMC5340865; doi:10.1128/mSystems.00199-16)
Supplement: TEXT S1 [file sys002172095s1.docx]

**Supplemental**

**Materials and methods**

**Supplemental references**

**Materials and methods**

*sOTU picking using Deblur*:

The Deblur algorithm (https://github.com/biocore/deblur) was run independently on each experiment using default parameters, in negative filtering mode, with a read length of 150bp. Taxonomy was added to the resulting BIOM (1) tables using the RDP (ribosomal database project) classifier (2) through QIIME (3). Deblur of the complete AGP data, and subsequent removal of blooms, took 3.25 hours of CPU time (45 minutes’ wall time on a 32 core node). sOTUs with less than 100 counts total were removed from the analysis; these cannot represent bloom sequences based on the mean log fold change criteria.

*Calculation of sOTU fold-change in storage studies*

Fresh frozen or freshly sequenced samples at time 0 were used as the base for comparison with a second time point (either 1, 4, 7, or 14 days at room-temp).

The fold-change in relative abundance was calculated for each sOTU for each individual, with the fold-change calculated as the mean over all individuals in which that sOTU was detected. Those sOTUs at a relative abundance less than 0.05% were rounded to 0.05% in order to reduce large fold-change due to multinomial sampling error.

*Calculation of sOTU fold-change in shipped vs. fresh frozen studies*

For each sOTU detected in the American Gut cohort, the mean fold-change between American Gut (shipped) and fresh frozen studies (UK Twins, Whole Grain Feces, and PGP) was determined by calculating the ratio of the mean (over all samples) between American Gut and each fresh frozen comparison study. For sOTUs comprising <0.01% of total reads in a sample, the abundance was treated as 0.01% (the low threshold was chosen as we are comparing means, which average the multinomial distribution).

*Selection of candidate blooming bacteria*

Analysis was performed only on bacteria showing a statistically significant difference (p-value = 0.0001) between AGP samples to all other fresh frozen experiments combined, using a permutation based mean log difference test with 10,000 permutations. The maximal fold-change over the 4 room temperature time points (compared to time 0) was used as the x-axis for each sOTU, thus indicating the maximal capability of each sOTU to grow at room temperature. Because the number of individuals participating in the storage studies is small, it’s possible that candidate blooming bacteria that were not present in the samples analyzed. In addition, due to effects resulting from compositionality, it is possible for this test to return false positives. Bacteria that appear to be growing may be the result of multiple other species that are declining, due to the dependence property induced from proportions (4). To account for this blooming bacteria were identified using the following criteria:

1. Proportion must increase more than two fold in the freeze thaw studies and the storage studies over time
2. Proportion must increase more than two fold in the freeze thaw studies, and the sOTU must be absent in all storage study samples.
3. Proportion must increase of more than 50 fold in the storage study

One important assumption made is that only a single or few bacteria might bloom in a sample.

To visualize blooming sOTUs, we plotted on the y-axis the minimal ratio between mean relative abundance levels in the American Gut cohort to the three fresh frozen studies. The minimal ratio over the three studies was used to mitigate per-study differences. We rationalized that if an sOTU was actually blooming, it would be higher in the American Gut cohort compared to all other studies, whereas if the difference in sOTU abundance was due to a factor specific to a single study (not affecting any other studies), the minimum fold-change would be close to 1.

*Visualizing the effect of blooming bacteria removal using PCoA*

A subset of 200 samples was randomly selected for the comparison of the American Gut and UK Twins studies. All fecal samples from the whole grain feces and PGP studies were also used (79 and 88 samples respectively). The sOTUs corresponding to the 10 candidate blooming bacteria were removed. Samples were rarified to 1000 reads/sample, the Bray-Curtis distance was calculated, and PCoAs were plotted and visualized using EMPeror (5).

*Determining the effect of blooming bacteria removal on taxonomy distribution*

The mean abundance of each bacterial class over all fecal samples was calculated before and after filtering the top 10 blooming sOTUs. Classes, other than Gammaproteobacteria, were sorted by frequency, and the top 7 phyla are shown. All additional classes were grouped into the “Other” class.

*Determining the effect of blooming bacteria removal on age-related alpha diversity correlation*

Fecal samples from the American Gut Project were obtained, and rarefied to a depth of 2000 sequences per sample. For each number of bloom sequences (0 to 20), a Kruskal Wallis test was used to compare age by decade in healthy adults (age 20-69, samples outside of that range were removed) to the Shannon diversity of the samples. The test statistic and the corresponding p-value are shown

*Code and data used for analysis*

Jupyter Notebooks (6) used for generating the figures and the datasets described in this manuscript are available at:

https://github.com/knightlab-analyses/bloom-analyses

**Supplemental references**

1. **McDonald D**, **Clemente JC**, **Kuczynski J**, **Rideout JR**, **Stombaugh J**, **Wendel D**, **Wilke A**, **Huse S**, **Hufnagle J**, **Meyer F**, **Knight R**, **Caporaso JG**. 2012. The Biological Observation Matrix (BIOM) format or: how I learned to stop worrying and love the ome-ome. Gigascience **1**:7.

2. **Wang Q**, **Garrity GM**, **Tiedje JM**, **Cole JR**. 2007. Naive Bayesian classifier for rapid assignment of rRNA sequences into the new bacterial taxonomy. Appl Environ Microbiol **73**:5261–7.

3. **Caporaso JG**, **Kuczynski J**, **Stombaugh J**, **Bittinger K**, **Bushman FD**, **Costello EK**, **Fierer N**, **Pena AG**, **Goodrich JK**, **Gordon JI**, **Huttley GA**, **Kelley ST**, **Knights D**, **Koenig JE**, **Ley RE**, **Lozupone CA**, **McDonald D**, **Muegge BD**, **Pirrung M**, **Reeder J**, **Sevinsky JR**, **Turnbaugh PJ**, **Walters WA**, **Widmann J**, **Yatsunenko T**, **Zaneveld J**, **Knight R**. 2010. QIIME allows analysis of high-throughput community sequencing data. Nat Methods **7**:335–336.

4. **Lovell D**, **Warren M**, **Taylor J**, **Zwart A**, **Helliwell C**. 2010. Caution ! Compositions ! Can constraints on omics data lead analyses astray ? Csiro.

5. **Vázquez-Baeza Y**, **Pirrung M**, **Gonzalez A**, **Knight R**. 2013. EMPeror: a tool for visualizing high-throughput microbial community data. Gigascience **2**:16.

6. **F Perez BRG**. 2007. IPython: a system for interactive scientific computing. Comput Sci Eng **9**:21–29.
